# N-type calcium channel and renal injury

**DOI:** 10.1007/s11255-022-03183-8

**Published:** 2022-04-13

**Authors:** Lei Bai, Shichao Sun, Yao Sun, Fujun Wang, Akira Nishiyama

**Affiliations:** 1 Department of Endocrinology, The Fourth Hospital of Hebei Medical University, No. 12 Jiankang Road, Shijiazhuang, 050011 Hebei People’s Republic of China; 2 Department of Neurology, The Second Hospital of Hebei Medical University, No. 215 Heping Road, Shijiazhuang, 050000 Hebei People’s Republic of China; 3 Department of Medical Image, The Fourth Hospital of Hebei Medical University, No. 12 Jiankang Road, Shijiazhuang, 050011 Hebei People’s Republic of China; 4 Department of Pharmacology, Kagawa University Medical School, Kagawa, 761-0793 Japan

**Keywords:** N-type calcium channel, Sympathetic nerve, Podocyte, Renal injury

## Abstract

Accumulating evidences indicated that voltage-gated calcium channels (VDCC), including L-, T-, N-, and P/Q-type, are present in kidney and contribute to renal injury during various chronic diseases trough different mechanisms. As a voltage-gated calcium channel, N-type calcium channel was firstly been founded predominately distributed on nerve endings which control neurotransmitter releases. Since sympathetic nerve is distributed along renal afferent and efferent arterioles, N-type calcium channel blockade on sympathetic nerve terminals would bring renal dynamic improvement by dilating both arterioles and reducing glomerular pressure. In addition, large body of scientific research indicated that neurotransmitters, such as norepinephrine, releases by activating N-type calcium channel can trigger inflammatory and fibrotic signaling pathways in kidney. Interestingly, we recently demonstrated that N-type calcium channel is also expressed on podocytes and may directly contribute to podocyte injury in denervated animal models. In this paper, we will summarize our current knowledge regarding renal N-type calcium channels, and discuss how they might contribute to the river that terminates in renal injury.

## Introduction

Voltage-gated calcium channels can be classified into L-, P/Q-, N-, R-, and T-type subtypes according their pharmacological and electrophysiological characters. In the kidney, a number of calcium channels comprising various α1 subunits, including Ca^2+^_V_2.1 (α1A), Ca^2+^_V_1.2 (α1C), Ca^2+^_V_1.3 (α1D), Ca^2+^_V_3.1(α1G), and Ca^2+^_V_3.2 (α1H), are expressed, and function as L-type (Ca^2+^_V_1.2, Ca^2+^_V_1.3), T-type (Ca^2+^_V_3.1, Ca^2+^_V_3.2), and P/Q-type (Ca^2+^_V_2.1) calcium channels. Furthermore, the kidney is supplied with numerous nerve endings that contain N-type (α1B) Ca^2+^ channels [1]. Recently we indentified N-type calcium channel expression in podocyte from both in vivo and in vitro experiments [2].

## N-type calcium channel in renal dynamic changes

Numerous studies have already reported that N-type voltage dependent calcium channels predominantly distributed in neuronal, especially sympathetic neuronal cells. These channels were intimately involved in sympathetic neurotransmission and regulated the release of norepineprine from sympathetic nerve endings [3–7]. This founding was clarified and supported by applying various N-type calcium channel antagonists in both in vivo [8–11] and clinical researches [12–14]. Underlying mechanisms could be simply summarized as follow: Calcium influx which mainly through voltage gated calcium channels in nerve endings will interact with soluble NSF attachment protein receptor (SNARE) proteins on synaptic vesicle and nerve terminal membranes. This interaction in turn causes exocytosis of neurotransmitters (e.g. NE) from the vesicles [15]. Although some neurons elicited resistant to N-type calcium channel antagonists suggested co-existence of other type calcium channels [16], N-type calcium channel still been believed as the main channel in mediating calcium influx in sympathetic nerves [3]. Abnormal activations of sympathetic nerve which innervated renal afferent and efferent arterioles have been indicated to play an important role in renal injury [17, 18]. Morphological studies clearly showed marked narrowing of afferent and efferent glomerular arterioles in the kidney during renal sympathetic nerve stimulation [19]. The insufficient oxygen and nutrient supply which due to significant reduced renal blood flow may underlie this renal dynamic injury. In contrast, renal denervation was verified by demonstrating a 50% decrease in renal norepinephrine spillover which resulted in a long-term reduction in arterial pressure[20]. Moreover, L-type calcium channel blockade causes predominant dilation of afferent arteriole. During hypertension, it will transmit systemic high blood pressure to kidney which potentially results in glomerular injury [21], whereas, N-type calcium channel inhibition decreases glomerular pressure by dilating both afferent and efferent arterioles [22].

**Fig. 1:**
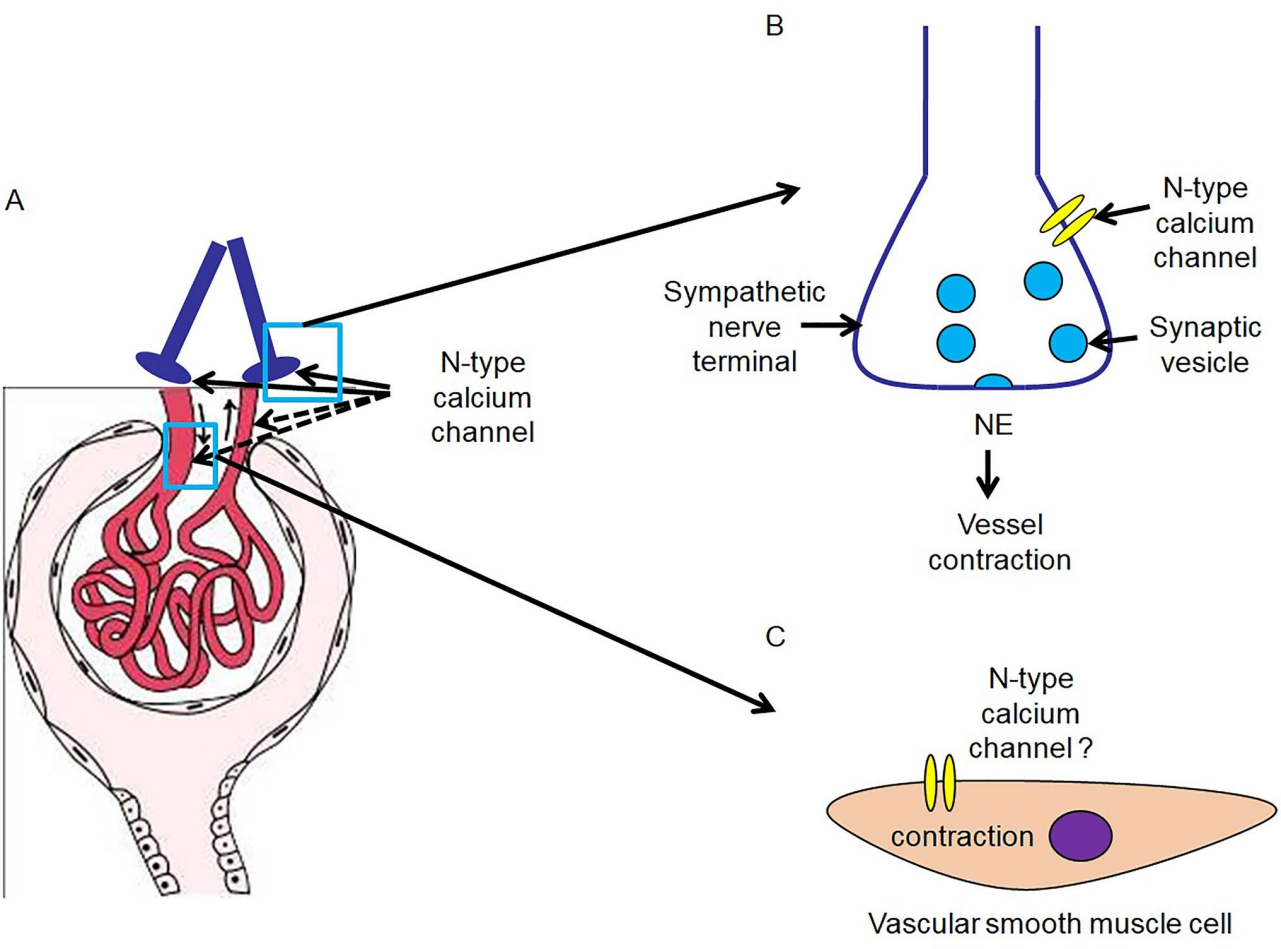
Involvement of N-type calcium channel in renal dynamic changes (**A**). N-type calcium channel may be involved in renal dynamic modulation through two pathways: 1. Calcium influx which mainly through N-type calcium channels in nerve endings will causes exocytosis of neurotransmitters (e.g., NE) from the vesicles thereby induces contraction of both afferent an efferent arteries (**B**). 2. N-type calcium channels may also directly mediate contraction of afferent and efferent arteries by inducing calcium influx into vascular smooth muscle cells (**C**). NE: Norepinephrine

Since excitation–contraction coupling in most resistance vessels is largely dependent on calcium influx through voltage-dependent calcium channels in rat kidney [23, 24], and local expressions of L- and T- type calcium channels already have been identified in smooth muscle cells which isolated from preglomerular vessels [25]. It is widely accepted that L- and T-type calcium channels are responsible for renal dynamic changes by controlling dilation or contraction of afferent and efferent arterioles. However, one issue raised since long time ago: L- and T-type VDCCs cannot fully account for calcium influx in renal vascular smooth muscle cells [26]. *Hansen et.al.* indicated the possible involvement of neuro-type calcium channel (e.g., P-/Q-type) in depolarization-mediated contraction in renal afferent arterioles [27]. Recently, both gene and protein expression of N-type calcium channel has been identified in dog basilar artery smooth muscle cells by Nikitina et al. [28], suggesting that, in addition to neural control, N-type calcium channel may also directly contribute to contraction of renal vessels by mediating calcium influx in vascular smooth muscle cells (Fig. 1).

## N-type calcium channel in renal non-dynamic changes

Besides renal vascular effects, N-type calcium channel has also been proved involve in non-dynamic changes during renal damage. An activated sympathetic nervous system was often characterized in chronic kidney disease, end-stage renal disease as well as diabetic nephropathy [29–33]. Therefore, neurotransmitter, such as norepinephrine, has been indicated as a mediator of sympathoexcitation induced renal injury by triggering some fibrotic and inflammatory signaling pathways in kidney. Reno-protective effects of N-type calcium channel inhibition were considered, at least partially, depend on inhibiting norepinephrine releases and thereby interfere with the fibrotic and inflammatory signaling pathways [9].

### N-type calcium channel and renal rennin-angiotensin system (RAS)

Cilnidipine showed superior effect in preventing proteinuria in hypertensive patients when compared with amlodipine [34–36]. These evidences from clinical trials suggested the unique contribution of N-type calcium channel blockade to renal injury should be independent of blood pressure control. Thus people try to figure out how does N-type calcium channel inhibition contribute to renal protection and elucidate possible involved mechanisms. Renin-angiotensin system is one of the hottest pathways may underlie this process.

Recent years, local RAS has been demonstrated as major role in pathogenesis of renal injury rather than circulating RAS. In the kidney, all of the RAS components are present and modulated by independent multiple mechanisms. For example, angiotensin II, the most powerful biologically active product of the RAS has been found be differently regulated in organs. In particular, the Ang II contents in renal tissues are much higher than can be explained on the basis of equilibration with the circulating concentrations [37–39]. This locally produced Ang II induces inflammation, cell growth, mitogenesis, apoptosis, migration, and differentiation, regulates the gene expression of bioactive substances, and activates multiple intracellular signaling pathways, all of which might contribute to tissue injury [40].

Since direct measurements of the intrarenal RAS components or micropuncture investigations in human subjects are not available, we may find our answers by applying in vivo studies. Cilnidipine treatment elicited significant stronger inhibition on albuminuria glomerular hypertrophy and interstitial fibrosis in dahl rats. In contrast, L-type calcium channel blocker, amlodipine did not show any effect on these parameters. In addition, urinary norepinephrine excretion, renal expression of renin mRNA and renal tissue levels of angiotensin II were increased only in the amlodipine-treated group [41]. Cilnidipine has been demonstrated more effective than, L-type calcium channel blocker amlodipine for preventing kidney injury in dahl rats [21, 41, 42]. This effect cannot be only explained by the L-type calcium channel blocking action that lowered blood pressure, but can be partially explained by the N-type calcium channel blocking action that lead to suppression of the sympathetic nerve activity and renal renin-angiotensin system.

Consistent founding has been reported by Toba, H. Glomerulosclerosis and collagen deposition in the tubulointerstitial area was significant attenuated by cilnidipine administration in DOCA-salt hypertensive rats. Importantly, accompany with these reno-protections, the renal activity and expression of angiotensin-converting enzyme (ACE) and the aldosterone concentration were inhibited by cilnidipine as well. However, these renal changes have not been observed in amlodipine treated group [43].

Activation of the renal renin-angiotensin system in diabetic patients always appears to contribute to diabetic nephropathy. Previously we investigated effects of cilnidipine on type 2 diabetic nephropathy by using SHR/ND rats. Vehicle treated group showed markedly increased urinary protein compared with healthy controls. Significant increased Ang II content and angiotensinogen mRNA expression were also detected in kidney. After 20 weeks treatment, cilnidipine significantly inhibited proteinuria, renal Ang II content and angiotensinogen mRNA expression. However, amlodipine did not elicit any effects on these parameters, despite anti-hypertensive effect [2]. Again, this founding suggested N-type calcium channel blockade may contribute to reno-protection by inhibiting the activated renal RAS in diabetic nephropathy. Moreover, renal AT1R has been reported to be significantly elevated in chronic pathogenesis which was believed associated with excessive renal sympathetic nerve activity. Renal denervation decreased this renal AT1R overexpression [44]. The possible relation of renal sympathetic nerve and RAS was suggested in in vitro study as well; Wang et.al. demonstrated that exogenous norepinephrine stimulates the expression of the AGT gene in the renal proximal tubule and which thereby increases the formation of local renal Ang II [45].

Taken together, renal RAS was inappropriately activated and play a critical role in renal injury during chronic renal diseases, hypertension and diabetic nephropathy. N-type calcium channel blockade may inhibit local RAS through, at least partially through, its neural control in kidney. The possible underlie mechanism can be summarized as follow: During pathological conditions, N-type calcium channel on renal sympathetic nerves was activated for mediating calcium influx which in turn triggered neurotransmitter release (e.g., norepinephrine). This elevated renal norepinephrine can induce upregulation of components of RAS in kidney lead to increase of angiotensin II, the major effective molecule of RAS which ultimately contribute to renal injury. N-type calcium channel inhibition can attenuate the inappropriate activation of RAS by preventing norepinephrine releases from sympathetic never in kidney.

However, local RAS activation cannot be fully explained by neural control. Our recent study suggested that N-type calcium channel blockade induced inhibition on renal RAS may independent of renal sympathetic nerve. In that study both renal norepinephrine and Ang II are significantly elevated in innervated vehicle treated-SHR rats. However, we also clearly demonstrated that cilnidipine significantly inhibited renal Ang II in renal denervated spontaneous hypertensive rats (SHR). In contrast, amlodipine did not show any effect on renal Ang II. Moreover, renal denervation just slightly decreased renal Ang II level in vehicle treated SHR, suggested two things: (1) Cilnidipine induced inhibition on renal RAS should be attributed to its N-type calcium channel blocking action. (2) However, this inhibition could be independent of renal sympathetic nervous system. There should be some other pathways have also been involved in N-type calcium channel mediated activation of renal RAS. Recent in vitro studies have already demonstrated existence of local RAS which can be activated by high glucose or mechanical stretch in various renal cells including mesangial cells [46, 47], proximal tubular cells [48–51], especially in podocytes [52–54]. During past decades, as a critical role in development of proteinuria and glomerulosclerosis, podocytes injury became a hot topic in research field of renal damage. We previously demonstrated that upregulation of N-type calcium channels in podocytes is company with significant elevated renal Ang II in diabetic nephropathy and hypertensive animal models. As indicated by Nitschke R, Ang II significantly increased intracellular calcium activity in podocytes via AT1 receptor. However, L-type calcium channel blocker, nicardipine, failed to inhibit this intracellular calcium activity, suggested that this AT1R mediated activation of intracellular calcium activity may be dependent on other calcium channels. In addition of identified N-type calcium channel on podocyte, we found exogenous Ang II induces significant increase of N-type calcium channel mRNA expression in cultured podocytes.

**Fig. 2:**
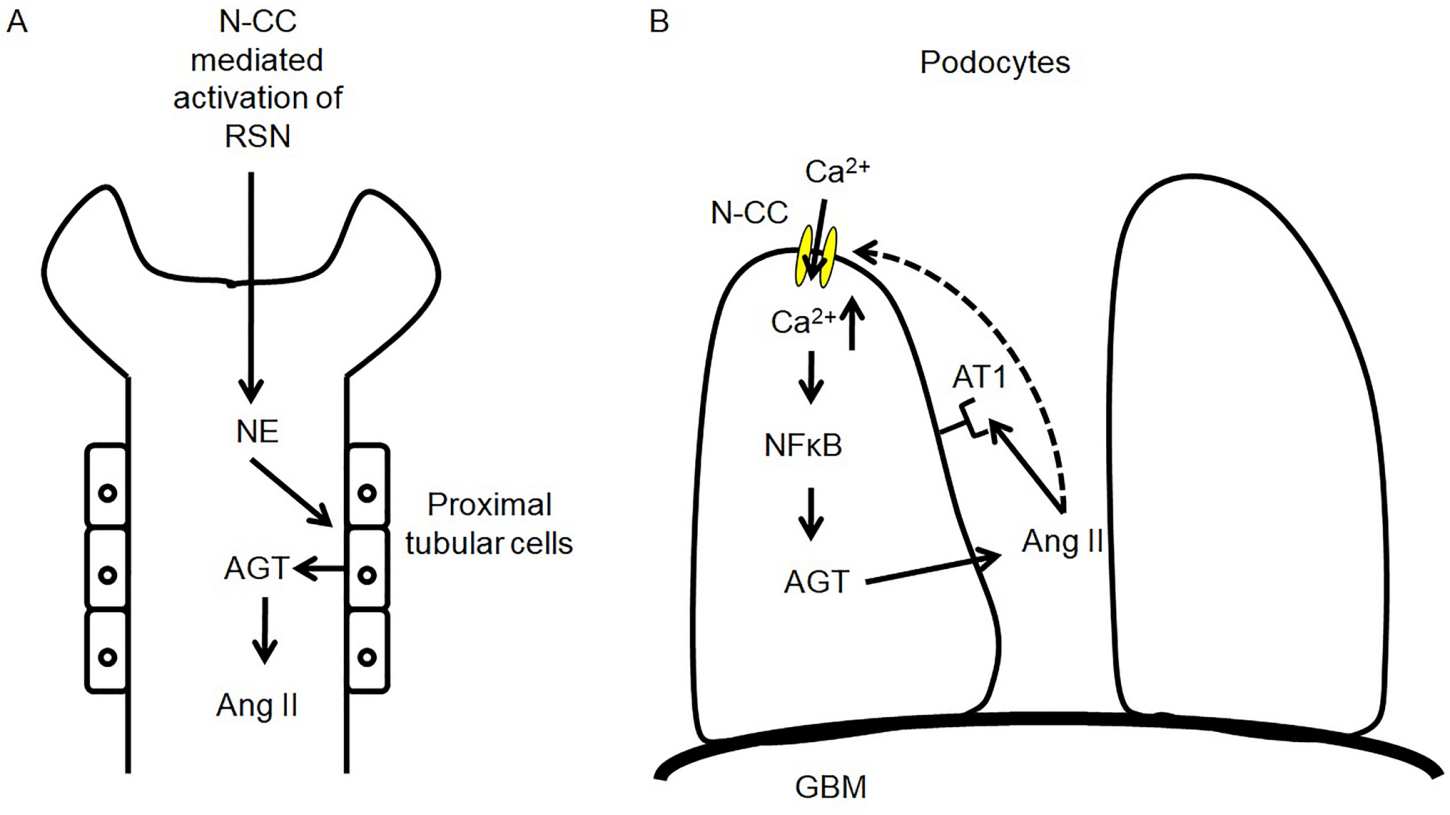
Relation between N-type calcium channel and renal RAS. Neuronal role of N-type calcium channel in renal RAS: Release of NE which was mediated by N-type calcium channel on renal sympathetic nerve terminals induces AGT production from proximal tubular cells. Since all components of RAS exsist in kidney, increase of AGT will finally contribute to production of Ang II (**A**). Non-Neuronal role of N-type calcium channel in renal RAS: N-type calcium channel mediated intracellular calcium increase triggers NFκB which may cause AGT synthesis and ultimately induce Ang II production. In addition, this elevated Ang II may further increase expression of N-type calcium channel on podocytes and formed vicious cycle (**B**). RSN: renal sympathetic nerve; AGT: angiotensinogen; Ang II: angiotensin II; AT1: angiotensin II type 1 receptor; N-CC: N-type calcium channel; NFκB: Nuclear factor κB

Nuclear factor κB (NFκB) has been suggested be activated and play a critical role in renal damage during hypertension and diabetes in both in vivo and in vitro studies [55–59]. Augmented intracellular calcium concentration has been indicated for mediating activation of NFκB signaling pathway in proximal tubular cells [60]. Moreover, as a transcriptional factor, NFκB has also been shown to modulate the rat and human AGT gene expression [61–63]. Since almost all components exist in podocyte [52], elevated AGT synthesis may finally lead to increase of Ang II production. Therefore, combined with our previous research, there may be a positive feedback between Ang II and N-type calcium channels. Activation of N-type calcium channel could be both consequence and cause of evoked renal RAS. Some factors, such as NFκB, maybe involved as parts or mediators of this vicious loop (Fig. 2).

### N-type calcium channel and renal oxidative stress

Among various factors involved in renal injury, oxidative stress, the imbalance of pro- and anti-free radical processes and the formation of excessive free radicals, also attracted enormous attention [64, 65].

Remarkable increase of renal reactive oxygen spices (ROS) production has been reported in different hypertensive [66–69] and diabetic [70–73] animal models via a nicotinamide adenine dinucleotide phosphate (NADPH) oxidase-dependent manner [74]. Accumulating evidence from both in vivo and in vitro studies further elucidated specific contribution of ROS overproduction to renal injury by targeting different cell types: Oxidant-mediated injury to tubular cells was suggested to play a critical role in tubulointerstitial fibrosis [75–78]. High glucose induces proliferation and extracellular matrix (ECM) synthesis of mesangial cells through activating NADPH oxidase which thereby cause ROS production [79–81]. In addition, mitochondrial dysfunction of mesangial cells also been reported could be induced by oxidative stress during high glucose condition [82]. Protein kinase C, mitogen-activated protein (MAP) kinase and NFκB have been implicated in the ROS overproduction induced abnormalities of mesangial cells [81, 83, 84]. In glomerular hypertension, where mechanical strain induced activation of extracellular signal-regulated protein kinases (ERK) was implicated in ECM deposition [85, 86]. Yatabe et.al. demonstrated mechanical strain induces phosphorylation of extracellular signal-regulated protein kinases (ERK) which mediated by activating NADPH oxidase in mesangial cells [87]. Continuous results also demonstrated that mechanical strain induced martrix production in mesangial cells through activating RhoA requires NADPH oxidase-mediated ROS generation [88]. Podocyte injury has also been associated with overactivation of ROS. High glucose induced podocyte hypertrophy has been implicated through a ROS-dependent activation of ERK1/2 and Akt/PKB [89]. Oxidative stress mediated podocyte apoptosis via activating p38 mitogen-activated protein kinase and caspase 3 has been suggested in cultured podoctyes and db/db mice by Susztak et al*.*[90]. Thus, numerous studies focused on renal antioxidant therapies for preventing renal damage during past decades.

Since greater antiproteinuric effect of cilnidipine was reported by Fujita et.al. in CARTER study when compared with amlodipine [36], people try to elucidate possible mechanism which underlie how N-type calcium channel blockade contribute to renal protection. Late results from same group showed that cilnidipine elicited significantly higher antioxidant activity than amlodipine and this superior antioxidant activity of cilnidipine has been proposed, at least in part, for explaining greater antiproteinuric effect [91]. In previous study, we analyzed renal TBARS content and DHE staining as oxidative stress markers in cilnidipine treated SHR/ND rats. Cilnidipine, but not amlodipine, significantly inhibited the increase in TBARS content and DHE staining. In addition, administration of cilnidipine suppressed the increase in mRNA levels of both gp91phox and p22phox, whereas amlodipine had no effect on expression levels. Protein complex formation of p47phox or Rac-1 with p22phox of NADPH oxidase subunits, which are necessary for NADPH oxidase to produce superoxide [92], were significantly increased in SHR/ND. Cilnidipine significantly suppressed the increases in complex formation of p47phox or Rac-1 with p22phox of NADPH oxidase. In contrast, amlodipine did not affect these parameters at all. These founds suggested again, N-type calcium channel played a critical role in renal ROS production which is dependent on NADPH oxidase. The precise mechanism about how does N-type calcium channel contribute to renal ROS production is still controversial.

Catecholamines, such as norepinephrine, can induce oxidative damage in myocardium through reactive intermediates resulting from their auto-oxidation [93]. Renal sympathetic activation been proposed to induce oxidative stress and lead to oxidative injury in end-organs such as the kidney [94–96]. Recently we also proved that renal denervation significantly suppressed aortic regurgitation induced glomerular reactive oxidative stress (ROS) [97]. Therefore, N-type calcium channel may activate oxidative stress by inducing norepinephrine releases from renal sympathetic nerve terminal.

A growing body of evidence from clinical and experimental studies has indicated role of RAS in induction of oxidative stress in the kidney [98, 99]. Results from 66 type 2 diabetic patients of nephropathy showed Treatment with an ARB for 8 weeks reduced the levels of urinary 8-epi-prostaglandin F2-α and 8-hydroxydeoxyguanosine, biochemical markers of oxidative stress [98]. Treatment of Wistar-Kyoto rats (WKY) with subcutaneous Ang II infusions from osmotic minipumps induced oxidative stress in association with increased expression of the p22phox component of NADPH oxidase and decreased expression of extracellular superoxide dismutase in the renal cortex [99]. Upregulation of protein and mRNA expressions of renal p47phox and iNOS were significantly attenuated by candesartan in type 2diabetic mice [100]. Thus N-type calcium blockade induced inhibition on renal oxidative stress may also be explained by reduction of local Ang II production.

**Fig. 3:**
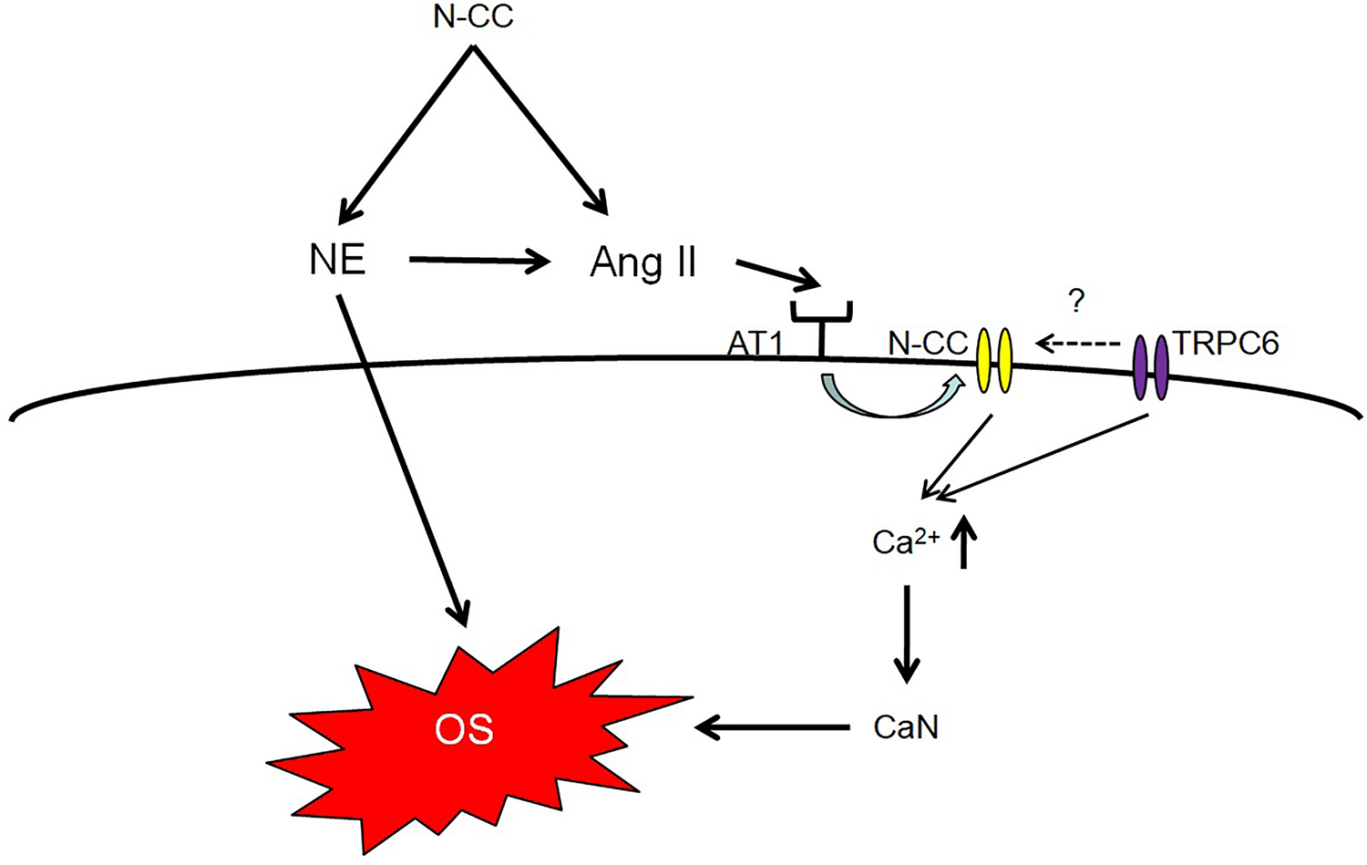
Relation between N-type calcium channel and oxidative stress. N-type calcium channel mediated production of NE and Ang II will cause oxidative stress on renal cells. In addition, N-type calcium channel may also involve in intracellular oxidative signaling pathway by inducing calcium influx and subsequent activation of calcineurin (Fig. 3). NE: Norepinephrine; Ang II: angiotensin II; AT1: angiotensin II type 1 receptor; N-CC: N-type calcium channel; CaN: calcineurin; TRPC6: transient receptor potential channel 6.; OS: oxidative stress

Interestingly, our recent in vitro study showed exogenous Ang II increases DHE staining in cultured mice podocytes. This increase can be significantly attenuated by knock down N-type calcium channel [2], suggesting that besides paracrine action, N-type calcium channel also directly involved in intracellular signal transduction pathway in Ang II induced oxidative stress. Indeed, it has been proved that calcium influx can trigger activation of calcium-dependent protein phosphatase calcineurin and its substrate nuclear factor of activated T cells (NFAT) in podosytes [101]. Off note, EI Bekay et al. demonstrated Ang II induced intracellular signal for ROS synthesis is transduced, at least partially, through calcium-dependent signaling pathway [102]. Although some studies associated calcium influx in podocytes to transient receptor potential (TRP) channels, especially TRPC6, we could not exclude the possibility that other sources, such as VDCCs, also be responsible for increase of intracellular calcium. As indicated by Nijenhuis, knock down of TRPC6 resulted in significant reduction of 1-oleoyl-2-acetylsn-glycerolin (OAG)-stimulated calcium influx in cultured podocyte. However, calcium influx was not completely inhibited, suggested involvements of other channels [101]. Actually, a secondary activation of L-type calcium channel was reported to be caused by Ang II induced TRPC3/C6 activation in cardiac myocytes [103]. Although L-type calcium channel has been suggested was not involved in Ang II stimulated calcium influx in rat podocyte since long time ago [104]. To our knowledge, there were still no studies identified L-type calcium channels in podocyte. The inefficient effect of L-type calcium channel blocker on preventing calcium influx probably due to nearly undetectable expression of L-type calcium channel in podocyte. In contrast, highly expressed N-type calcium channel was identified by our group in both in vitro and in vivo experiments [2]. Taken together, N-type calcium channel activation can trigger renal oxidative stress by inducing norepinephrine release and local Ang II synthesis which ultimately contribute to renal damage. In addition, N-type calcium channel may also directly involve in intracellular signal transduction pathway of Ang II induced oxidative stress (Fig. 3).

## Conclusion

Since been reported predominantly distributed in neuronal cells, especially sympathetic neuronal cells, the physiological and pathological role of N-type calcium channel in kidney were always tend to be mainly explained by its neural control during past decades. Indeed, N-type calcium channel was involved in modulating renal vascular tone and trigger some inflammatory and fibrotic signaling pathways through mediating neurotransmitter, such as norepinephrine, releases from renal nerve terminals. However, accumulating evidence from denervated animal models and in vitro studies emerges that N-type calcium channel was also expressed on renal cells, especially podocytes, other than neuronal cells and make distinct contribution to renal damage. N-type calcium channel mediated calcium influx could be the critical factor for these intracellular signaling transduction pathways. Further studies still are needed for clarifying precise mechanism for applying N-type calcium channel blockade as new clinical strategy for preventing renal damage.
